# The Value of the Laryngoscope

**Published:** 1866-05

**Authors:** G. Bruhl

**Affiliations:** Cincinnati, Ohio


*The Value of the Laryngoscope.*—By G. BRUHL, M. D., Cincinnati, Ohio.

Among the valuable additions of modern times to our means of exact diagnosis and treatment, the laryngoscope holds a very prominent place, and with certainty may we predict, that ere long no practitioner will be considered a good diagnostician who does not understand its use as well as that of the stethoscope or uterine speculum. Surely it is not necessary that every physician should acquire the dexterity of a specialist; not necessary that every one should be able to cauterize ulcers of the ary-eppiglottidean folds, remove foreign bodies from the hyoid fossa, or extirpate morbid growths from the vocal cords; but *it is necessary* that every one shall become sufficiently master of its use to be able to detect at least these pathological conditions and avoid the blunder of the distinguished London professor, who sent his patient, a young lady who had been aphonic for several years, to the country, to breathe the fresh air of her native place, and have the mortification afterwards of learning that some specialist had cured her by the removal of a polypus from the vocal cords.

True, the application of the laryngeal mirror is somewhat difficult for the beginner; but is the art of auscultation and percussion easier to be learned? Months, sometimes, will pass away before the student understands the meaning of the physical signs. In laryngoscopy, a little skill and energy will overcome in less time the presenting obstacles, and the labor bestowed for this purpose rewards richly. Wonderful are the results, and he who has once seen through the rima glottidis of a sufferer, fed on cod liver oil and hypophosphites *ad nauseum*, and who had detected there, not tubercular deposits as supposed, but a morbid growth as the real materia peccans, best knows how to appreciate the great value of this diagnostic apparatus.

Bringing a hitherto hidden cavity under our direct observation, the laryngoscope has not only corrected our physiological views of the functions of the different parts of the larynx and pharynx in the act of phonation and deglutition, but it has, what is of greater importance to the practitioner, taught us to interpret accurately the symptoms of the larygeal diseases, and has enriched and enlarged our knowledge of their pathology and treatment by giving us new and direct indications, and guiding our operative manipulations.

We do not need to fill up our symptomatology with mere guess work, or form our ideas by philosophical—often erroneous—deductions and speculations. Those morbid changes, of which we can find no trace on the dissection table, or if any, only in the last stages, we can now observe from the beginning to the end. We see now exactly every part which is affected, see the size, the seat, the shape, the extension of the morbid processes and growths; can even explain the causes why certain parts by their anatomical and physiological condition are more liable to certain diseases than others; we know what parts of the larynx are pre-selected by certain diseases for their morbid deposits and destructions, and why they are; nay, even we can distinguish the different forms of the morbid changes of the epiglottis as well as those of the glottis muscles.

And this is of peculiar importance in the treatment of aphonia; for experience has shown that in such cases, where aphonia is merely dependent upon paralysis of the glottis muscles, the electric current effects an infallible cure. Why, we do not understand at present; time may clear the secret, but the assertions of renowned laryngoscopists are too numerous and too positive to allow a doubt in the truth of their statement.

The superior usefulness of the laryngoscope, however, is best shown in its being the only reliable means of detecting the morbid growths in the larynx. All the signs and symptoms heretofore attributed to them are uncertain; either they can be wanting according to the size or seat of the tumors, or they can be assimilated by other diseases. Therefore the grossest diagnostic errors have been committed; the most irrational modes of treatment instituted; therefore so many lives unnecessarily been lost. Wherever the doctor came across a patient with hoarseness, aphonia and troublesome cough, he was sure to feed him on cod-liver oil or send him to some watering place, there to be cured from his supposed laryngeal phthisis or catarrh. But hardly one thought of morbid growth within the larynx.

In prelaryngoscopical times, therefore, not more than eighty cases of this kind have been reported, and most of them were only detected upon *post mortem* examination. In five cases only were successful operations performed. No wonder: the diagnosis being uncertain, nobody could tell which cases would be favorable for surgical aid.

But since the few years of the discovery of the laryngoscope, the history of these growths has undergone a total change. More than a hundred new cases have been reported and successfully operated upon. A precise aid for the diagnostician's eye and a sure guide for the operator's hand, the laryngoscope has made success certain, where it has been heretofore but accidental. Cauterization of ulcers, opening of abscesses, removal of excrescences, scarification of hypertrophied parts, can now be practiced with the same preciseness, though with more tediousness, within the larynx, than on the outside surface.

Even in such cases, in which by laryngoscopic examinations only a negative result is obtained, we are at least taught that we have misinterpreted the symptoms, and that we have to seek for the real seat of the disease elsewhere than in the larynx.

Semeleder gives several very interesting cases of this kind, of which I will relate but one. He was called to examine the throat of a lady, apparently dying from suffocation. The most careful examination had revealed nothing positive; the attending physician, suspecting some trouble in the larynx, had concluded to perform tracheotomy to relieve her from her sufferings. The laryngoscope did not detect any abnormal condition, but as there existed a difference between the radial pulses, the doctor declared an aortic aneurism as the real case of the dyspnœa, and the operation useless and uncalled for. The attack passed away, and the symptoms becoming subsequently more distinct, the correctness of his diagnosis was corroborated. The lady soon died, but she died without an unnecessary operation. The laryngoscope saved her at least the addition of a new suffering to her old one.

Czermak relates a similar case in his *Der Kehlkopf Spiegel*, page 107. A lady was about to be operated upon for a supposed stenosis of the larynx. The instruments lay spread on the table, when the surgeon, a distinguished one, cautious enough, sent for the laryngoscopist, to ascertain the correctness of the diagnosis. Well, the larynx was found in a healthy condition, of normal size, without any obstruction. These cases are of peculiar interest, as they demonstrate clearly that laryngoscopic examinations can give positive contra-indications against *tracheotomy*—perhaps better against useless operations, to which the surgeon, deceived by the symptoms, otherwise might be misled.

Now, if in the face of these facts any one doubts the value of the laryngoscope, or ridicules its practicability, he is not its, he is his own enemy. Not every one can be a genius; not every one advance new ideas or invent new instruments, but every scientific physician should be prudent enough to profit from practical discoveries.—*Cincinnati Lancet & Observer.*

